# Modern treatment outcomes for early T-stage oropharyngeal cancer treated with intensity-modulated radiation therapy at a tertiary care institution

**DOI:** 10.1186/s13014-020-01705-1

**Published:** 2020-11-10

**Authors:** Eric J. Di Gravio, Pencilla Lang, Hugh Andrew Jinwook Kim, Tricia Chinnery, Neil Mundi, S. Danielle MacNeil, Adrian Mendez, John Yoo, Kevin Fung, Joe S. Mymryk, John W. Barrett, Nancy Read, Varagur Venkatesan, Sara Kuruvilla, Lucas C. Mendez, Eric Winquist, Sylvia Mitchell, Sarah A. Mattonen, Anthony C. Nichols, David A. Palma

**Affiliations:** 1 Department of Otolaryngology – Head and Neck Surgery, Western University, London, ON Canada; 2 Department of Oncology, Western University, London, ON Canada; 3 Department of Medical Biophysics, Western University, London, ON Canada; 4 Department of Microbiology and Immunology, Western University, London, ON Canada; 5 Department of Otolaryngology - Head and Neck Surgery, Victoria Hospital, London Health Science Centre, 800 Commissioners Road East, London, ON N6A 5W9 Canada

**Keywords:** Radiation, Chemoradiation, Oropharyngeal cancer, Toxicity

## Abstract

**Background:**

Transoral surgery (TOS), particularly transoral robotic surgery (TORS) has become the preferred modality in the United States for the treatment of early stage oropharyngeal cancer, largely due to assumptions of fewer toxicities and improved quality of life compared to primary radiotherapy (RT). However, these assumptions are based on retrospective analysis, a subset of which utilize primary RT groups not limited to T1-2 stage tumors for which transoral robotic surgery is FDA approved. Thus, there is potential for underestimating survival and overestimating toxicity, including treatment related mortality, in primary RT.

**Methods:**

Consecutive cases of early T-stage (T1–T2) oropharyngeal cancer presenting to the London Health Sciences Centre between 2014 and 2018 treated with RT or chemoradiation (CRT) were reviewed. Patient demographics, treatment details, survival outcomes and toxicity were collected. Toxicities were retrospectively graded using the Common Terminology Criteria for Adverse Events criteria.

**Results:**

A total of 198 patients were identified, of which 82% were male and 73% were HPV-positive. Sixty-eight percent of patients experienced a grade 2 toxicity, 48% a grade 3 and 4% a grade 4. The most frequent toxicities were dysphagia, neutropenia and ototoxicity. The rates of gastrostomy tube dependence at 1 and 2 years were 2.5% and 1% respectively. There were no grade 5 (fatal) toxicities. HPV-positive patients experienced improved 5-year overall survival (86% vs 64%, p = 0.0026).

**Conclusions:**

Primary RT or CRT provides outstanding survival for early T-stage disease, with low rates of severe toxicity and feeding tube dependence. This study provides a reference for comparison for patients treated with primary transoral surgery.

## Background

Over the past few decades, there has been a dramatic rise in the incidence of oropharyngeal squamous cell carcinoma (OPSCC), largely due to increasing rates of infection with human papillomavirus (HPV) [1]. HPV-associated OPSCC patients tend to be younger and healthier than traditional smoking- and alcohol-related OPSCC patients, and have a significantly improved prognosis. The 5-year survival of HPV-associated OPSCC exceeds 80%, making quality of life after treatment increasingly important since these patients may now survive for many years with significant treatment toxicities [2]. This has led to intense interest in treatment de-escalation, with the goal of reducing the toxicity of standard dose chemoradiation therapy [3]. In the United States, transoral surgery (TOS), particularly transoral robotic surgery (TORS), has largely become the preferred treatment modality for early T-stage OPSCC, as retrospective data has suggested a more favourable toxicity profile [4]. However, there is a paucity of data directly comparing the two modalities making the choice of treatment for OPSCC highly controversial [5].

The drive to adopt TOS as the standard of care revolves around the assumption that it carries a lower toxicity profile, and therefore better quality of life, than standard chemoradiation [5]. The ORATOR study, a phase II trial of 68 patients, is the only randomized clinical trial to directly compare quality of life between primary radiotherapy and primary TOS approaches, and included patients regardless of HPV status [6]. In this trial, toxicity profiles differed between the two modalities. In particular, swallowing-related quality of life as measured by the MD Anderson Dysphagia Inventory was statistically superior in the chemoradiation group as compared to the TORS group, although the difference did not represent a clinically meaningful change [6].

This discrepancy with previously published retrospective data can partially be explained by two potential sources of bias in previous studies: the inclusion of advanced stage OPSCC in chemoradiation cohorts, as well as improved toxicity profiles of modern radiation techniques [6, 7]. TORS is only FDA approved for treatment of early stage (T1-2) OPSCC. However, many retrospective studies comparing TORS to chemoradiation include chemoradiotherapy cohorts containing advanced T-stage disease even though these patients are generally not considered candidates for TORS [4, 8–11]. Furthermore, advances in radiotherapy such as intensity-modulated radiation therapy (IMRT) allow for more conformal treatment plans, reducing prevalence and severity of side effects such as dysphagia [12–14].

The purpose of this study is to examine the modern outcomes and toxicity in patients with early stage disease (T1-2, N1-2) treated with IMRT ± chemotherapy at a high-volume tertiary care cancer centre. We include only patients with T1-2 N1-2 disease as these patients would be considered candidates for treatment with primary transoral laser or robotic surgery.

## Methods

### Study participants and clinical features

Research Ethics Board approval (17222E) was obtained. A retrospective chart review was performed of all patients with early stage OPSCC treated with curative-intent radiotherapy ± chemotherapy who presented to the London Health Sciences Centre (LHSC) between 2014 and 2018. Early stage was defined as American Joint Committee on Cancer 7th Edition stage T1-T2, N0-N2. Patient, tumour and treatment-related factors collected included: gender, age at diagnosis, smoking and alcohol history, site of primary tumour (base of tongue, palatine tonsil, soft palate, vallecula, lateral or posterior pharyngeal wall, or unknown), TNM stage, HPV status, and use of concurrent chemotherapy. HPV status was determined with p16 immunohistochemical analysis with strong and diffuse staining in > 70% of tumour cells considered positive. Patients are reported with the chemotherapy protocol that they initiated treatment with regardless of whether they completed the full course or were switched to a different protocol during treatment. Alcohol abuse was defined as a history of > 20 alcoholic beverages per week. A significant smoking history was defined as a total of 10 or more pack-years.

### Radiotherapy

All patients received definitive intent radiotherapy with IMRT with fixed-gantry or rotational techniques (tomotherapy or volumetric modulated arc therapy [VMAT]). Patients generally received 70 Gy in 35 fractions (5 daily fractions delivered per week) to the gross disease, and 56 Gy in 35 fractions to the elective nodal volume. Patients were generally treated with concurrent high dose cisplatin (100 mg/m^2^ given every three weeks), excluding patients aged ≥ 70, those with comorbidities or poor performance status or those who declined. Weekly cisplatin (40 mg/m^2^) could be used at the discretion of the medical oncologist. If patients were not suitable for concurrent cisplatin, alternative chemotherapy regimens could include the Calais regimen (carboplatin and 5-fluorouracil) or cetuximab. If patients did not receive concurrent systemic therapy an accelerated fractionation (6 fractions per week delivered over 5 days) was used at the discretion of the treating oncologist. Unilateral radiation was used for tonsil primaries with less than 1 cm extension into the tongue base or palate with ≤ 1 ipsilateral lymph node. All other patients received bilateral treatment.

### Evaluation and follow-up

After completion of radiotherapy all patients were seen at 6 weeks for a clinical assessment of response and treatment toxicity, every 3 months for the first 2 years, and then every 4 to 6 months in the third to fifth years. A follow-up computed tomography (CT) scan was obtained at 3 months, with additional imaging, including positron emission tomography-computed tomography (PET-CT), if there was an incomplete response or if clinically indicated. Salvage surgery was reserved for patients who had persistent disease on follow-up imaging or clinically suspicious findings. All recurrences were histologically confirmed if possible.

### Toxicity assessment

Treatment toxicities were graded using the CTCAE version 5.0 [15]. Toxicities or death occurring during or within 30 days of the end of treatment were included. Toxicities assessed included dysphagia, neutropenia, febrile neutropenia, ototoxicity and acute kidney injury. Any use of nasogastric, gastrostomy (G), gastrostomy-jejunostomy (GJ) tube or total parental nutrition (TPN) within this time frame was considered a grade 3 dysphagia. Any change in diet not requiring the aforementioned interventions was considered grade 2 dysphagia.

### Statistical analysis

Descriptive statistics were generated for baseline patient characteristics for all patients (n = 198). Overall survival (OS) and disease-free survival (DFS) were calculated from the date of treatment to date of recurrence (DFS), date of death, or date of last follow-up, whichever occurred first. Kaplan-Meier estimates were generated for OS and DFS stratified by HPV status and compared using the log-rank test. Univariable and multivariable Cox proportional hazards regression  was performed for both OS and DFS to assess the association between baseline characteristics and OS and DFS respectively. Backward step-wise analysis was used to create the final multivariate model of overall and disease-free survival (Additional files 1 and 2). All statistical analysis was performed using the R language environment for statistical computing version 3.4.0 (open source, www.r-project.org), using two-sided statistical testing at the 0.05 significance level.

## Results

### Patient and treatment characteristics

**Fig. 1:**
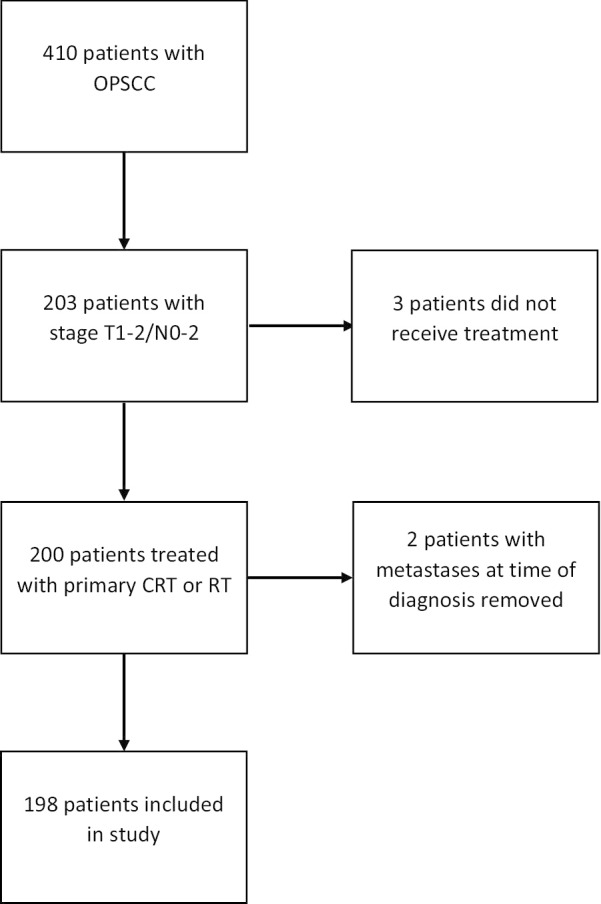
Flowchart of oropharyngeal cancer patients. CRT—chemoradiation; OPSCC—oropharyngeal squamous cell carcinoma; RT—radiotherapy

A total of 198 patients (163 male and 35 female) treated with chemoradiation or radiotherapy alone were included in this analysis. Figure 1 depicts a flowchart of the screening process for inclusion. The crude median follow-up for surviving patients was 27.4 months from the completion of treatment (range: 0–63). Baseline and treatment characteristics are summarized in Table 1. Median age at diagnosis was 61 years with 114/198 patients (57.6%) having greater than a 10 pack-year smoking history and 49/198 (24.7%) consuming more than 20 alcoholic beverages per week. One hundred and forty-four of 198 (72.7%) patients had proven p16 positive disease and 140/198 (70.7%) and 125/198 (63.1%) had stage T2 and N2 disease respectively. One hundred and fifty-nine of 198 (80.3%) patients were treated with chemoradiation with the most common chemotherapy protocol being monotherapy with cisplatin. Of patients receiving cisplatin, 61.4% received high dose treatment, while 38.5% received weekly treatment (Table 1). Of patients receiving high dose cisplatin, 70/80 (87.5%) received 2 or more cycles with 54/80 (67.5%) completing all three. Of patients treated with weekly cisplatin, 45/55 (81.8%) completed 5 or more cycles with only 17/55 (30.9%) completing all 7 cycles.

**Table 1:** Baseline and treatment characteristics

	All patients (n = 198)
Age, median (IQR)	61 (54–66)
Sex	
Male	163 (82.3%)
Female	35 (17.7%)
Total pack-years	
< 10 pack-years	84 (42.0%)
≥ 10 pack-years	114 (57.6%)
Alcohol consumption	
< 20 drinks per week	149 (75.3%)
≥ 20 drinks per week	49 (24.7%)
Primary site	
Tonsil	108 (54.5%)
Base of tongue	76 (38.4%)
Soft palate	9 (4.5%)
Vallecula	3 (1.5%)
Indeterminate	2 (1.0%)
Clinical T-stage	
T1	58 (29.3%)
T2	140 (70.7%)
Clinical N-stage	
N0	21 (10.6%)
N1	52 (26.3%)
N2	125 (63.1%)
p16 status	
Positive	144 (72.7%)
Negative	28 (14.1%)
Unknown	26 (13.1%)
Treatment	
RT	39 (19.7%)
CRT	159 (80.3%)
Radiation laterality	
Unilateral	14 (7.1%)
Bilateral	163 (82.3%)
Unknown	21 (10.6%)
CT simulation	
With contrast	85 (42.9%)
Without contrast	92 (46.5%)
Unknown	21 (10.6%)
Chemotherapy regimen	
Cisplatin	135 (68.2%)
High dose	83 (61.4%)
Weekly	52 (38.5%)
Carboplatinum + 5-fluorouracil	14 (7.1%)
Cetuximab	5 (2.5%)
Other	5 (2.5%)

Data are presented as number (%) unless otherwise specified

*RT* radiotherapy only, *CRT* chemoradiation

### Treatment toxicity

Data on acute toxicity is summarized in Table 2. One hundred and thirty five of 198 (68%) of patients experienced at least one grade 2 toxicity, 95/198 (48%) a grade 3 toxicity and 8/198 (4%) a grade 4 toxicity. Of note, no patients experienced a grade 5 (fatal) toxicity during treatment. The most common toxicity was dysphagia followed by neutropenia and ototoxicity. Patients treated with radiation alone experienced fewer toxicities compared to patients treated with chemoradiation (p < 0.0001). Most notably, in total, 56/198 (28%) of patients experienced grade 3 dysphagia and 37/198 (19%) required placement of G/GJ tube. However, the rate of G/GJ tube use during treatment was significantly greater in patients treated with chemoradiation compared to patients treated with radiation alone (p = 0.0196 ). Among patients treated with radiation alone, rates of grade 3 dysphagia and G/GJ tube insertion during treatment were 4/39 (10%) and 2/39 (5.1%) respectively, compared to 52/159 (33%) and 33/159 (21%) for patients treated with chemoradiation. Two patients (1 treated with radiation alone and the other with chemoradiation) required G/GJ tube placement more than a month after completion of treatment. In total, the 1- and 2-year rates of G/GJ tube use were 2.5 and 1% respectively (5 and 2 patients respectively). Only 8 patients (4%) experienced a grade 4 toxicity (2 ototoxicity, 6 neutropenia).

**Table 2:** Summary of toxicities

	Radiotherapy group (n = 39)				Chemoradiation group (n = 159)			
Toxicity	Grade 1–2	Grade 3	Grade 4	Grade 5	Grade 1–2	Grade 3	Grade 4	Grade 5
Dysphagia	26 (67%)	4 (10%)	0	0	99 (62%)	52 (33%)	0	0
Neutropenia	0	0	0	0	64 (40%)	34 (21%)	6 (4%)	0
Febrile neutropenia	0	0	0	0	0	14 (9%)	0	0
Ototoxicity	1 (3%)			0	46 (29%)	34 (21%)	2 (1%)	0
Acute kidney injury	0	0	0	0	0	10 (6%)	0	0

Data are presented as numbers (%). Grading is consistent with Common Terminology Criteria for Adverse Events (CTCAE) version 5.0

### Predictors of overall and disease-free survival

In total, 32/198 (16%) patients included in this study died. Seventeen of these 32 (53%) patients died with disease still present, either as a direct result of their disease or due to unrelated causes, while 15/32 (47%) died of unrelated causes while in remission. Furthermore, 32/198 (16%) of patients experienced disease recurrence, of which 27/32 (84%) were histopathologically proven. Nine of 32 (28%) recurrences were local, 13/32 (41%) were regional, 8/32 (25%) were distant, 1/32 (3%) was both local and regional and 1/32 (3%) was both regional and distant. Nineteen of 32 (59%) patients with recurrence required salvage surgery.

**Fig. 2:**
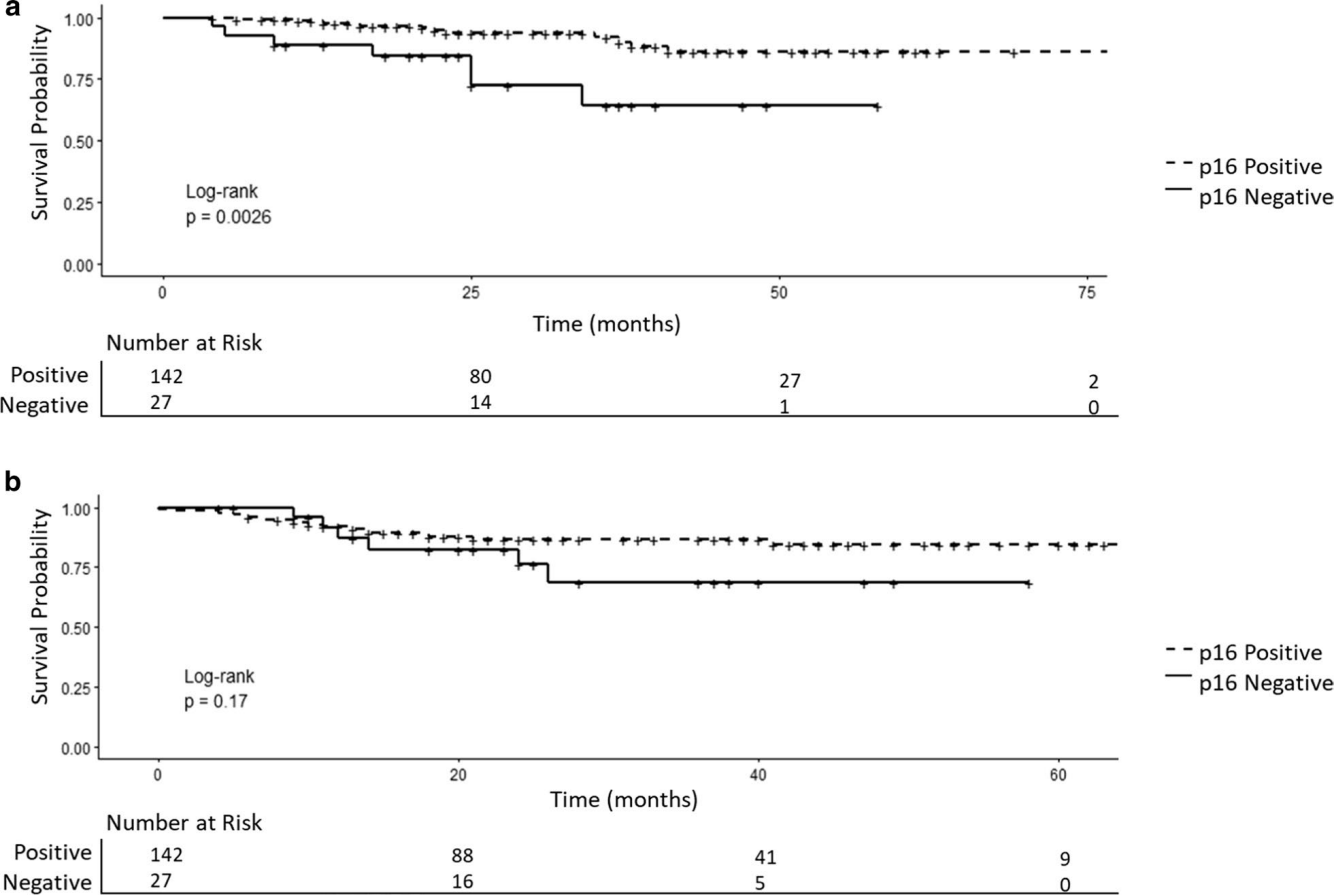
Overall (**a**) and disease-free (**b**) survival for all patients with early-stage OPSCC seen at the London Health Sciences Centre by p16 status (27 patients p16 negative; 142 patients p16 positive; 26 patients with unknown status were excluded)

Thirty-two of 198 patients with incomplete clinical information were omitted from Cox proportional hazards regression. Univariable analysis revealed age, smoking status, alcohol use and p16- status as significant prognostic factors for OS (Additional file 1). With multivariable analysis, smoking history (HR: 3.59 95% CI: 1.02–12.7, p = 0.0472) remained a significant prognostic factor for OS (Additional file 1). Age and alcohol use were predictors of DFS in univariable and multivariable analysis (Additional file 2). p16-positive status was associated with improved OS (p = 0.0026) but not DFS (p = 0.17, Fig. 2).

### Patterns of treatment failure

**Fig. 3:**
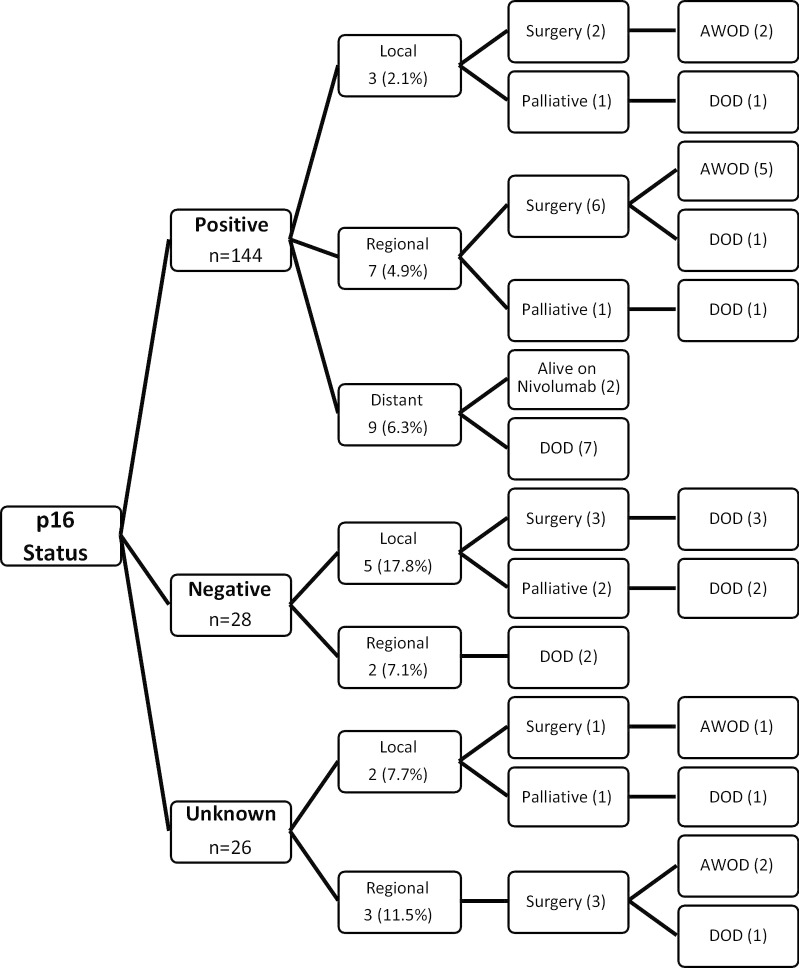
Patterns of treatment failure stratified by p16 status (positive, negative or unknown). AWOD—alive without disease; DOD—dead of disease

Disease relapse stratified by p16 status is outlined in Fig. 3. Consistent with the literature, p16-negative patients were more likely to experience locoregional relapse than p16-positive patients (7/28 vs. 10/144, p < 0.01), and significantly more likely to die from that recurrence (7/7 vs. 3/10, p=0.0089) [3]. There were nine distant metastatic failures in the p16-positive cohort including lesions in the lung, liver and skeleton, while none occurred in the p16-negative group, however this was not statistically significant (9/144 vs. 0/28, p = 0.36).

## Discussion

In this study, we demonstrated that while IMRT-based (chemo)radiation can have short- and long-term toxicities, early-stage HPV-associated OPSCC patients experienced excellent survival with acceptable toxicities, low long-term gastrostomy dependency rates and negligible treatment-related mortality (0% in this study). In contrast, prior studies have reported higher toxicity rates, with treatment related deaths of up to 3% [2, 16]. Similarly, a frequently cited meta-analysis of the Radiation Therapy Oncology Group treatment intensification trials by Machtay and colleagues reported that 43% of patients treated with primary chemoradiation for OPSCC suffered a severe major late toxicity, including a 10% rate of long-term feeding tube dependency [16]. This is relevant as these studies are often cited as a reference comparison for primary transoral surgery [4, 8–11]. While TOS can be carried out for advanced stage disease, in most case series, > 85% of tumours are limited T1-T2 stage [4, 8–10]. In contrast, the chemoradiation studies included high rates of advanced T stage disease (> 75% in one such study by Ang and colleagues) [3], introducing bias into the comparison of historical series. Furthermore, many of these studies were carried out in the pre-IMRT era, which would likely impact function and toxicity [2, 13, 14, 16]. Patients eligible for TOS have low-volume disease that may allow for unilateral radiotherapy and improved normal tissue sparing compared to historical chemoradiation cohorts that include a wider range of patients. There is a deficiency in the literature of reported outcomes of solely early-stage patients. This study reports the outcomes and toxicities of early-stage oropharynx patients undergoing primary radiotherapy with modern techniques, and provides a historical comparison cohort for discussions around surgical outcomes. Rates of toxicity and treatment-related mortality are lower than in previously published cohorts.

Febrile neutropenia is a potentially life threatening and frequent complication of chemoradiation in many studies [17, 18]. For example, a study by Bledsoe and colleagues reported a rate of febrile neutropenia of 26% in patients treated with chemoradiation with 2/32 (6.3%) of these patients dying as a direct result [17]. In contrast, 19.7% of our early-stage patients did not require chemotherapy at all and thus were not at risk of this complication. In the chemoradiation cohort specifically, the rate of febrile neutropenia was only 8.8% and there were no fatalities (Table 2).

One of the strongest predictors of poor patient quality of life following treatment for head and neck cancer is long-term gastrostomy tube dependence [19]. Chemoradiation studies report rates of up to 10% long-term dependence while most TOS studies show lower rates [4, 20, 21]. In a previous systematic review, the majority of studies demonstrated gastrostomy rates of less than 4.5%, with many (6/13) showing rates of 0% suggesting superior swallowing function with primary surgery [4]. However, this study has 2-year gastrostomy rates of 1%. This is similar to the results of the ORATOR trial, and suggest that swallowing outcomes are similar between the two treatment strategies [6]. Given the similar survival, treatment selection for early OPSCC should be an informed decision made between clinicians and patients.

In this study, we also attempt to describe potential prognostic indicators for OS and DFS. It is well-established that HPV-associated OPSCC has a significantly better OS and DFS than HPV-negative patients [3]. In our study, OS but not DFS was found to be significantly better in HPV-associated OPSCC, possibly due to limited sample size (Fig. 1). In early stage disease, chemoradiation appeared effective regardless of HPV status, and the differences in OS may be partially related to the presumed increased incidence of comorbidities in HPV-negative patients and subsequent non-cancer related death. Likely for similar reasons, on multivariable analysis, a history of smoking was associated with a worse OS but not DFS (Additional files 1 and 2).

Concurrent chemoradiation with 3 cycles of high-dose cisplatin is currently the standard treatment for locoregionally advanced OPSCC [22]. However, due to short- and long-term toxicities, and due to the fact that HPV-associated OPSCC is more sensitive to chemotherapy and radiation than HPV-negative OPSCC, there has been much interest in treatment de-escalation [23, 24]. Some current strategies under investigation include weekly cisplatin instead of high-dose cisplatin, lower radiation dose, or decreased adjuvant radiation and/or chemotherapy after surgery [25–27]. Other strategies, such as using the epidermal growth factor receptor (EGFR) monoclonal antibody cetuximab, have conclusively been shown to provide inferior survival without meaningfully improving quality of life which only further highlights the importance of balancing toxicity with survival [28, 29].

There are a number of limitations with this current study. First of all, it is inherently difficult to accurately grade toxicities retrospectively, precluding the inclusion of other common but less severe toxicities such as mucositis, xerostomia or peripheral neuropathy. The follow-up period was limited and thus may have led to an underestimation of long-term toxicities. This data is limited to a single centre and thus may not accurately portray the true variability in the patient population. Lastly, a number of important potential confounders such as socioeconomic factors and compliance with treatment were not addressed.

## Conclusions

Patients with early-stage OPSCC have excellent survival, and current assumptions about the toxicities associated with chemoradiation are likely over-stated in this population. Early-stage patients have low rates of gastrostomy tube dependence and treatment-related mortality. The lower rate of toxicity in early-stage patients compared to historical series is important to keep in mind when comparing toxicity profiles between chemoradiation and TOS. This study can provide a reference for comparison for patients treated with primary transoral laser or robotic surgery in future trials.

## Supplementary information

**Additional file 1.** Univariable and multivariable analysis of overall survival in all patients with early stage disease seen at the London Health Sciences Centre from 2014 to 2018 by clinical characteristics. Samples missing clinical information were excluded (30 patients were excluded, 168 remaining samples in the analysis). Backwards step-wise method was utilized to arrive at the final multivariate model that was based on patient age, alcohol abuse, and smoking status. P-values < 0.05 are bolded. HR—hazard ratio; CI—confidence interval; BoT—base of tongue; HPV—human papillomavirus.

**Additional file 2.** Univariable and multivariable analysis of disease-free survival in all patients with early stage disease seen in the London Health Sciences Centre from 2014 to 2018 by clinical characteristics. Samples missing clinical information were excluded (32 patients were excluded, 166 remaining samples in the analysis). Backwards step-wise method was utilized to arrive at the final multivariate model that was based on patient age, sex, and alcohol abuse. P-values < 0.05 are bolded. HR—hazard ratio; CI—confidence interval; BoT—base of tongue; HPV—human papillomavirus.

## Data Availability

All data generated and analyzed during this study are included in this published article (and its supplementary information files).

## Abbreviations

CRT: Chemoradiation
CTCAE: Common terminology criteria for adverse events
DFS: Disease free survival
HPV: Human papillomavirus
IMRT: Intensity modulated radiotherapy
LHSC: London Health Sciences Centre
OPSCC: Oropharyngeal squamous cell carcinoma
OS: Overall survival
RT: Radiotherapy
TORS: Transoral robotic surgery
TOS: Transoral surgery
VMAT: Volumentric modulated arc therapy
