# Supplementary material for: Modern treatment outcomes for early T-stage oropharyngeal cancer treated with intensity-modulated radiation therapy at a tertiary care institution
Source: Radiat Oncol. 2020 Nov 10;15:261. doi: 10.1186/s13014-020-01705-1 (PMC7654053; doi:10.1186/s13014-020-01705-1)
Supplement: Supplementary file 1 — Additional file 1. Univariable and multivariable analysis of overall survival in all patients with early stage disease seen at the London Health Sciences Centre from 2014 to 2018 by clinical characteristics. Samples missing clinical information were excluded (30 patients were excluded, 168 remaining samples in the analysis). Backwards step-wise method was utilized to arrive at the final multivariate model that was based on patient age, alcohol abuse, and smoking status. P-values < 0.05 are bolded. HR—hazard ratio; CI—confidence interval; BoT—base of tongue; HPV—human papillomavirus. [file 13014_2020_1705_MOESM1_ESM.docx]

**Additional File 1.**

| \|  \|  \| **Univariate** \| \| **Multivariate** \| \| \| --- \| --- \| --- \| --- \| --- \| --- \| \| **Variables** \| \| **HR (95% CI)** \| **P Value** \| **HR (95% CI)** \| **P Value** \| \| **Age** \|  \| 1.06 (1.01 - 1.11) \| **0.025** \| 1.06 (0.99 - 1.11) \| 0.056 \| \| **Sex** \| Male Vs. Female \| 1.76 (0.40 - 7.64) \| 0.453 \|  \|  \| \| **Smoking** \| Smoker Vs. Non-Smoker \| 4.83 (1.39 - 16.70) \| **0.013** \| 3.59 (1.02 - 12.70) \| **0.047** \| \| **Alcohol abuse** \| Yes Vs. No \| 3.11 (1.23 - 7.85) \| **0.016** \| 2.25 (0.88 - 5.77) \| 0.091 \| \| **Subsite** \| Other Vs. BoT \| 0.62 (0.078 - 4.88) \| 0.648 \|  \|  \| \| Tonsil Vs. BoT \| 0.52 (0.20 - 1.35) \| 0.179 \|  \|  \| \| **T stage** \| T2 Vs. T0-T1 \| 0.99 (0.37 - 2.66) \| >0.99 \|  \|  \| \| **N stage** \| N2 Vs. N0-N1 \| 0.75 (0.28 - 2.04) \| 0.579 \|  \|  \| \| **HPV status** \| Positive Vs. Negative \| 0.24 (0.090 - 0.61) \| **0.003** \|  \|  \| |
| --- | --- | --- | --- | --- | --- | --- | --- | --- | --- | --- | --- | --- | --- | --- | --- | --- | --- | --- | --- | --- | --- | --- | --- | --- | --- | --- | --- | --- | --- | --- | --- | --- | --- | --- | --- | --- | --- | --- | --- | --- | --- | --- | --- | --- | --- | --- | --- | --- | --- | --- | --- | --- | --- | --- | --- | --- | --- | --- | --- | --- | --- | --- | --- | --- | --- |
